# Enhancing adult neurogenesis attenuates hippocampal-related behavioral deficits in an Alzheimer’s mouse model

**DOI:** 10.3389/fnins.2026.1833016

**Published:** 2026-06-22

**Authors:** Chi-Chieh Lee, Federico Calegari

**Affiliations:** Center for Regenerative Therapies Dresden (CRTD), Technische Universität Dresden, Dresden, Germany

**Keywords:** 3xTg-AD mouse model, adult hippocampal neurogenesis, Alzheimer’s disease, hippocampal behavior, neural stem cell

## Abstract

Alzheimer’s disease (AD) is the most prevalent form of dementia, characterized by progressive memory loss, cognitive decline, and emotional dysregulation. Adult hippocampal neurogenesis (AHN) critically contributes to cognition and mood but undergoes precipitous decline during AD progression. Here, we investigated whether enhancing AHN through genetic expansion of endogenous neural stem cells (NSC) ameliorates AD-related phenotypes. Using lentiviral overexpression of the cell cycle regulators Cdk4 and CyclinD1 in the dentate gyrus of 3xTg-AD mice, we show that enhancing AHN is accompanied by partial improvements in selected behavioral measures associated with hippocampal function, including in the open-field test and Morris water maze. These findings indicate that the AD-compromised neurogenic niche remains responsive to NSC-targeted stimulation and support the use of AHN as a potential additional avenue for multi-modal therapeutic strategies for AD.

## 1 Introduction

Alzheimer’s disease (AD) is a progressive neurodegenerative disorder characterized by profound memory loss and cognitive decline. The pathological cascade underlying AD involves the accumulation of amyloid-beta (Aβ) plaques and tau tangles, leading to neuronal loss and disruption of neural networks that result in memory impairments and mood dysregulations (Breijyeh and Karaman, 2020; Knopman et al., 2021). The hippocampus, a critical hub for memory processing, spatial navigation, and emotional regulation (Burgess et al., 2002; Strange et al., 2014; Eichenbaum, 2017; Moser et al., 2017), represents a primary target of AD pathology and focal point for therapeutic intervention.

As a hallmark of the hippocampus, an endogenous reserve of neural stem cells (NSC) resides within the dentate gyrus (DG), serving as a source of adult-born neurons throughout life in a process referred to as adult hippocampal neurogenesis (AHN). AHN plays a pivotal role in maintaining the plasticity and adaptability of the hippocampal circuitry (Toda et al., 2019). Highlighting its multifaceted role, AHN not only positively contributes to learning and memory processes but is also linked to mood regulation (Anacker and Hen, 2017; Kempermann, 2022). These roles of AHN are particularly relevant, providing avenues to rescue deficits resulting from AD with significant clinical implications. However, as one of the earliest features of AD, AHN undergoes a precipitous decline during disease progression, raising concerns pertaining to its significance and therapeutic potential (Moreno-Jiménez et al., 2019; Salta et al., 2023).

Given AHN’s critical roles in cognition and mood, multiple strategies have emerged to enhance neurogenesis as a therapeutic approach for AD. These strategies include stimulation of endogenous AHN through physical exercise or other physiological stimuli (Choi et al., 2018; Valenzuela et al., 2020). While partially effective, these approaches could not discriminate between effects triggered by AHN within the DG versus any of the many systemic effects that the physiological stimuli themselves are known to trigger, including massive re-wiring of the whole hippocampal connectivity and activity (Emery et al., 2023). Alternative strategies to attempt to rescue neuronal loss included cell therapy interventions involving NSC transplantation (Si and Wang, 2021) or glia–neuron reprogramming (Zhou et al., 2022), which in either case was ineffective in the replacement of neurons undergoing physiological maturation, specification and functional integration. Recent molecular interventions utilizing microRNA or preventing neuronal death have provided stronger evidence for a role of AHN in the rescue of AD (Walgrave et al., 2021; Mishra et al., 2022). Nonetheless, additional proof-of-principles are important to validate the effects of enhancing endogenous NSC, without systemic effects or external cell sources, to restore hippocampal function in an AD-compromised neurogenic niche. This is critical for establishing AHN as a robust and reproducible therapeutic strategy for AD.

In this context, our group has previously developed a viral transduction approach based on the overexpression of the cell cycle regulators Cdk4 and CyclinD1 (together referred to as 4D) (Artegiani et al., 2011) that alone was sufficient to promote the expansion of endogenous NSCs. This approach resulted in the generation and functional integration of 2- to 3-fold higher number of newborn neurons in the mouse DG over the course of life and without exhaustion of NSC (Berdugo-Vega et al., 2020). Enhancement of AHN by 4D overexpression was shown to solely depend on a cell-intrinsic expansion of NSC, rather than secondary effects on mature neurons or glia (Artegiani et al., 2011) and resulting in the increased generation of newborn neurons that preserved their morphological, molecular and electrophysiological properties (Alonso et al., 2019; Berdugo-Vega et al., 2020). Ultimately, 4D-increased neurogenesis was accompanied by improvements in cognitive flexibility and memory indexing (Berdugo-Vega et al., 2021). It is noteworthy to mention, however, that our previous results were obtained in a healthy neurogenic niche. Whether 4D could still promote NSC expansion and attenuate behavioral deficits in the AD-compromised hippocampus is unknown. To this aim, here we investigated the efficacy of the 4D system in enhancing AHN within the 3xTg-AD mouse model and assessed its impact on hippocampal cognitive function.

## 2 Materials and methods

### 2.1 Animal handling

Mice were kept in standard cages with a 12-h light cycle with water and food ad libitum. Female, 6-month-old, 3xTg-AD mice with a mixed C57BL/6, 129×1/SvJ, 129S1/Sv genetic background were used in all experiments. Females were selected because in the 3xTg-AD line they show more severe AD pathology and behavioral deficits at an earlier age than males (Clinton et al., 2007; Carroll et al., 2010; Sterniczuk et al., 2010; Javonillo et al., 2022). Suspensions of lentiviruses, harvested from HEK293T cells transfected with p6nst-GFPloxpNLSloxp or p6nst-GFPloxpNLS4Dloxp vectors, in which expression is driven by the ubiquitin promoter (Artegiani et al., 2011), were prepared as previously described at a titer of 10^8–9^ IU/mL (Berdugo-Vega et al., 2020) and 1 μL bilaterally injected in the DG of isoflurane-anesthetized mice at a constant flow of 200 nL/min using a nanoliter-2000 injector (World Precision Instruments) and a stereotaxic frame Model 900 (Kopf Instruments) at ±1.6 mm mediolateral, −1.9 anterior–posterior, and −1.9 mm dorsoventral from bregma. In the present study, 4D expression was induced chronically without subsequent tamoxifen-mediated recombination of the 4D cassette aiming to trigger long-term increase in neurogenesis as previously described (Berdugo-Vega et al., 2020). To assess AHN, mice received intraperitoneal injections of BrdU (Sigma) dissolved in PBS at 50 mg/kg body weight, administered twice daily for 5 consecutive days beginning 8 weeks after surgery, with tissue analysis performed either 2 or 8 weeks after the first BrdU injection (i.e., 10 or 16 weeks post-surgery total). Animals were subjected to behavioral tests, anesthetized with xylazine/ketamine, and perfused transcardially with saline followed by 4% paraformaldehyde (PFA) fixation in phosphate buffer for immunohistochemistry. Animal procedures were performed according to local regulations (DD 24–9168.11-1/2011-11, TVV 45/2021).

### 2.2 Behavioral tests

Open field test (OFT) was performed in 60 × 60 cm field with 40 cm high white plywood walls. The room was illuminated at 300 lux (100 W light bulb installed 1.5 m above the apparatus). Each mouse was placed in one corner of the empty arena and allowed to freely explore for 10 min. The exploratory activity of the mice was recorded with a camera (Logitech) and analyzed with EthoVision software (Noldus) to assess distance, speed and time spent in the central area (inner 50% open field area). After each trial, the apparatus was cleaned with 75% ethanol to avoid odor cues. Morris Water Maze (MWM) was performed as previously described (Berdugo-Vega et al., 2020) in a circular pool (1.89 m diameter) filled with water kept at 19–20 °C and made opaque with a white non-toxic pigment. Mice that underwent both OFT and MWM were given one week of rest between tests. Mice were trained to find the square escape platform (9 × 9 cm) submerged 0.5 cm below the water surface over 8 days including 4-day learning and 4-day reversal. Each day, mice performed 4 trials with a minimum inter-trial time of 15 min. Each trial lasted a maximum of 2 min at the end of which mice were guided to the platform and then placed in individual drying cages equipped with a warm light. On the first day of reversal, the platform was moved to the opposite quadrant to assess re-learning. Data acquisition and analysis were performed with the EthoVision system (Noldus). Navigational strategies were assessed by unbiased automated analysis of swim trajectories using Rtrack, according to Overall et al. (2020). Each trial was assigned one predominant search strategy, as previously described (Garthe et al., 2009; Garthe and Kempermann, 2013). Strategies were grouped as follows: thigmotaxis reflected swimming along the pool wall; random strategies included circling, random search, and scanning; chaining reflected an egocentric search pattern at a relatively fixed distance from the wall; hippocampal/allocentric strategies included directed search, focal search, and direct swimming toward the platform; and perseverance was defined during the first day of reversal as preferential searching toward the previous platform location. Representative examples of each strategy are shown in Supplementary Figure 1A.

### 2.3 Immunohistochemistry

Brains were harvested after perfusion and post-fixed in 4% PFA at 4 °C overnight. After washing with PBS and embedding in 3% low-melting agarose, brains were coronally cut into 40 μm thick slices by a vibratome. Serial sections along the rostral-caudal axis of the hippocampus (every sixth section in one tube, total 6 tubes) were collected and stored in cryoprotectant solution (25% Ethanol and 25% glycerol in PBS) at −20 °C. For immunolabeling, slices were washed with PBS, blocked and permeabilized with 10% donkey serum in 0.3% Triton X-100 in PBS for 1.5 h at room temperature. Primary and secondary antibodies (Supplementary Table 1) were diluted in 3% donkey serum in 0.3% Triton X-100 in PBS and incubated overnight at 4 °C or 2 h at room temperature, respectively, with DAPI being added for the last 10 min. For BrdU or Aβ detection, slices were exposed to HCl 2 M for 25 min at 37 °C or 88% formic acid for 3 min at 70 °C before blocking, respectively. Pictures were acquired with Zeiss ApoTome with maximal intensity projections of three optical sections (10 μm total) or AxioScan microscopes (Carl Zeiss) followed by Extended Depth of Field Calculation.

### 2.4 Cell quantification

For each animal, cell quantification was performed using Photoshop CS5 (Adobe) on coronal hippocampal sections sampled at regular intervals along the rostral-caudal axis of the DG. Specifically, every sixth section from the serial section series was selected for staining and quantification. For each animal, 6–8 sections per brain were analyzed (except for 2 out of 31 samples for which only four sections were available due to tissue loss or damage). Viral transduction was evaluated by GFP expression. Quantifications were normalized to the infected GFP + population within each animal to account for minor variations in viral delivery between animals. Representative rostro-caudal images showing GFP + infected cells in the AD/4D DG are shown in Supplementary Figure 1C. For each animal, marker-positive cells were summed across the sampled DG sections. Unless otherwise specified, values were expressed as percentages using the summed number of GFP + infected cells from the same sections as the denominator. For short-term proliferation, BrdU+ cells were expressed as a percentage of Sox2 + GFP + cells. Each mouse contributed one percentage value and was considered one independent biological replicate. Aβ load was quantified with Fiji (ImageJ) as the percentage of Aβ-immunoreactive area relative to the total hippocampal area analyzed on stereotaxically equivalent coronal sections.

### 2.5 Statistical analysis

Cell and Aβ quantification were assessed on individual mice used as independent biological replicates (numbers indicated in the result and figure legend). Data are reported as individual point and mean ± SD (Supplementary Tables 2, 3) as indicated in the figure legends. For pairwise comparisons, Shapiro–Wilk tests were used to assess normality and F-tests were used to assess equality of variances. Student’s unpaired t-test, Welch’s unpaired t-test, or Mann–Whitney U test was then applied according to the outcome of these assumption tests. MWM performance across repeated trials or days was analyzed by two-way ANOVA. Navigational strategies were analyzed using Wald test of odds ratios assessed by logistic regression. Strategy change (%) = [(strategy proportion in comparison group − strategy proportion in reference group) / strategy proportion in reference group] × 100. AD/GFP was used as the reference group.

## 3 Results

### 3.1 4D overexpression restores AHN in 3xTg-AD mice

Our group has previously developed an approach to genetically increase the expansion of endogenous NSC by overexpression of the cell cycle regulators Cdk4 and cyclinD1 (together: 4D) (Artegiani et al., 2011). Promoting AHN, injection of 4D lentiviruses in the DG was found to improve cognitive function, navigational performance, memory precision and indexing over the course of life (Berdugo-Vega et al., 2020; Berdugo-Vega et al., 2021). However, since previous studies were performed in healthy mice, it is unknown whether the same 4D strategy could be used to increase AHN and improve hippocampal function in an AD-compromised neurogenic niche. More specifically, mouse models of AD are characterized by a major reduction in the number of NSC and impaired AHN (Mu and Gage, 2011; Salta et al., 2023). These deficits arise early in life, in juvenile mice, well before the onset of amyloid plaque formation and cognitive impairments (Liu et al., 2023). In turn, this indicates that AD underlies a highly dysfunctional neurogenic niche in which 4D may not be sufficient to overcome barriers to rescue deficits in NSC numbers, their proliferation and/or survival of newborn neurons.

**Figure 1:**
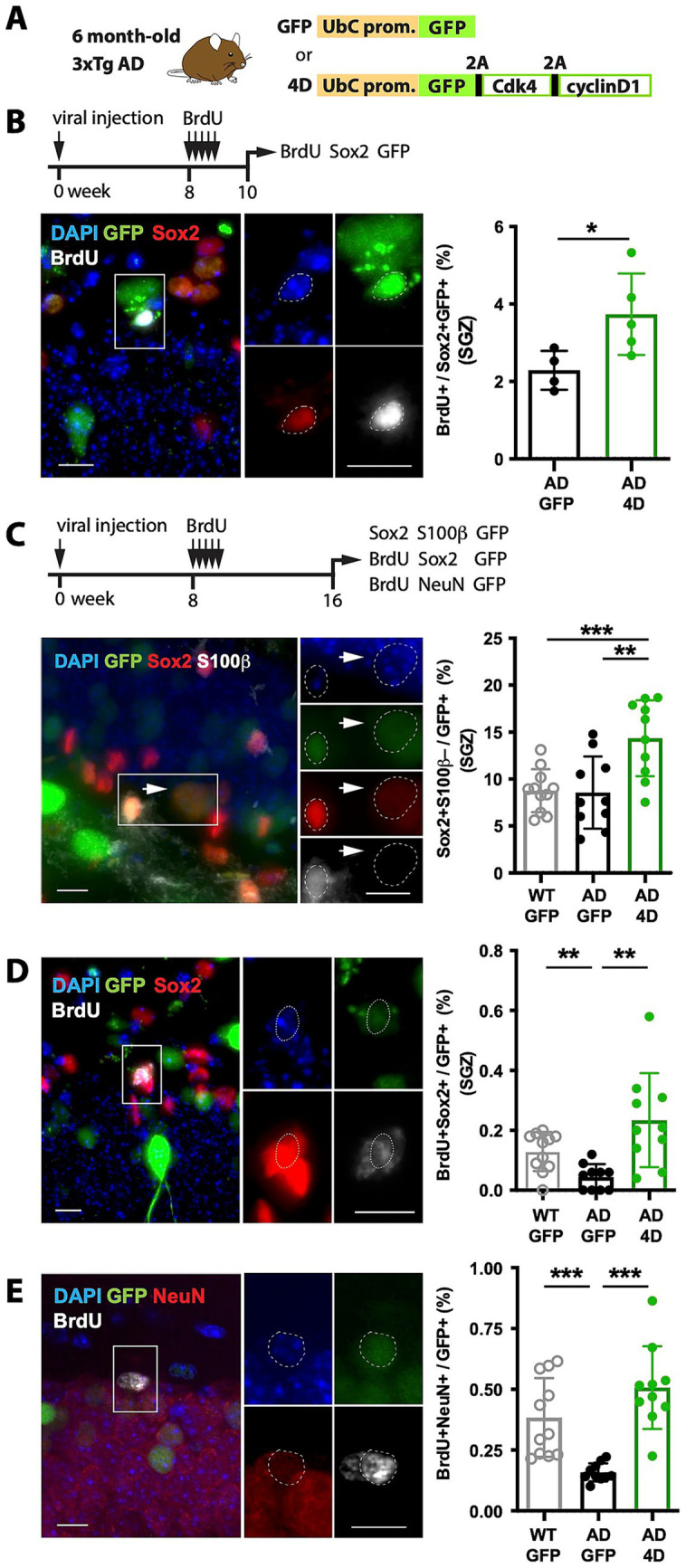
4D increases NSC expansion and neurogenesis in 3xTg-AD mice. **(A)** Schemes of GFP or 4D lentiviral constructs injected into the DG of 6-month-old female 3xTg-AD, or WT mice. **(B–E)** Experimental layout (top), representative fluorescence pictures (left) and quantifications (right) of cellular markers (as indicated) in the subgranular zone (SGZ) or the DG of mice injected with GFP or 4D viruses (as indicated). Insets indicate magnified regions, and examples of scored cells are indicated by arrowheads or dotted lines in brains. Cohorts of mice were sacrificed 10 weeks (**B**; *n* = 4 and 5 for AD/GFP, and AD/4D, respectively) or 16 weeks (**C–E**; *n* = 11, 10, and 10 for WT/GFP, AD/GFP, and AD/4D, respectively) after viral injection. Values are shown as bar graph with individual data points and mean ± SD, listed in Supplementary Table 2. Pairwise comparisons were performed using Student’s unpaired t-test, Welch’s unpaired t-test, or Mann–Whitney U test according to Shapiro–Wilk normality tests and F-tests for equality of variances. * *p* < 0.05; ** *p* < 0.01; *** *p* < 0.001. Scale bar = 10 μm.

To explore the efficacy of our 4D approach during the progression of AD, we used the 3xTg-AD mouse model. This model is widely used and closely resembles AD pathologies (Oddo et al., 2003), including cognitive deficits (Billings et al., 2005). Aligned with studies characterizing 3xTg-AD in female mice (Clinton et al., 2007; Carroll et al., 2010; Sterniczuk et al., 2010; Javonillo et al., 2022), we also observed cognitive deficits in the MWM arising at 6 months of age including reduced hippocampal/allocentric navigation during learning and increased perseverance upon reversal (change in probability of WT vs. AD: hippocampal/allocentric learning = 171%, perseverance = 313%; logistic regression, odds ratio (OR) of WT vs. AD: hippocampal/allocentric learning = 5.22, perseverance = 5.6; Wald-test, *p* < 0.0001 and *p* < 0.001, *n* = 8 and 6, respectively; Supplementary Figure 1A). Also aligned with studies using this AD mouse model, we observed increased hippocampal amyloid plaque burden at the final time point analyzed, around 10 months of age (immunoreactive area in hippocampus in healthy control and 3xTg-AD, mean ± SD: 0.05 ± 0.04% vs. 0.43 ± 0.22%, *p* < 0.01; *n* = 4 and 10 for WT/GFP and AD/GFP group respectively; Supplementary Figure 1B). Next, to investigate the possibility to increase the expansion of endogenous NSC, 4D stereotaxic viral injections were performed targeting the DG of 6-month-old 3xTg-AD mice (hereafter referred to as AD/4D). Mice underwent chronic NSC expansion with continuous 4D expression recapitulating our previous study (Berdugo-Vega et al., 2020). In parallel, age-matched wildtype or 3xTg-AD mice injected with GFP viruses were used as healthy or experimental controls, respectively (hereafter referred to as WT/GFP or AD/GFP; Figure 1A; top). In the three experimental conditions, representative sections across the rostro-caudal axis illustrated GFP + transduced cells distributed throughout the sampled DG (Supplementary Figure 1C) and neither AD/GFP nor AD/4D mice showed any change in amyloid plaque burden (mean ± SD: 0.43 ± 0.22% vs. 0.51 ± 0.2%, *p* = 0.41; *n* = 10 and 10, respectively; Supplementary Figure 1B).

To assess the efficacy of 4D overexpression in the dysfunctional AD niche, we first examined the proliferative responses of NSC by administering BrdU for 5 days at the end of an 8-week period of continuous 4D overexpression. At 10 weeks after viral injection, number of BrdU+ cells was expressed as a percentage of GFP+, infected cells expressing the stem/progenitor cell marker Sox2 (i.e.: BrdU+/Sox2 + GFP+). This revealed a ca. 60% increase in the proportion of proliferating infected cells in AD/4D relative to AD/GFP mice (AD/GFP vs. AD/4D: mean ± SD: 2.29 ± 0.5% vs. 3.73 ± 1.05%; *p* < 0.05; *n* = 4 and 5, respectively; Figure 1B), which was fully consistent with earlier quantifications in healthy mice (Berdugo-Vega et al., 2020; Berdugo-Vega et al., 2021).

Having confirmed the 4D effect on short-term proliferation, we next investigated whether this translated into long-term effects on parameters related to neurogenesis. As before, BrdU was administered for 5 days beginning 8 weeks after viral injection, but this time animals were sacrificed 8 weeks later. This paradigm was chosen to infer, among viral-transduced (GFP+) cells, indicators of (i) NSC abundance, (ii) BrdU long-term label retention within quiescent NSC, (iii) overall neurogenic output and/or neuronal survival. Because these markers were used to assess distinct biological readouts, each quantification was calculated using the appropriate denominator.

We first assessed NSC abundance by quantifying bona fide NSC, defined as Sox2 + S100β−, among infected GFP + cells (i.e.: Sox2 + S100β−/GFP+) irrespective of BrdU incorporation. Here, no statistically significant difference was observed between WT/GFP and AD/GFP mice (mean ± SD: 8.77 ± 2.29% vs. 8.56 ± 3.84%; *n* = 11 and 10 respectively; *p* = 0.88). In contrast, AD/4D mice showed a nearly 2-fold increase in the abundance of NSC (mean ± SD: 14.34 ± 4.04%; *n* = 10) relative to both WT/GFP and AD/GFP mice (*p* < 0.001 and *p* < 0.01 respectively; Figure 1C). This increase upon 4D overexpression corroborates and extends the previous effect on short-term proliferation described above (Figure 1B) and is fully consistent with previous reports in young and aged, healthy mice (Berdugo-Vega et al., 2020; Berdugo-Vega et al., 2021).

Having confirmed the 4D-effects on NSC proliferation and NSC abundance, we next investigated whether these changes were associated with an increase in the long-term reserve pool of quiescent NSC by assessing BrdU-label retention within infected cells (i.e.: BrdU+Sox2+/GFP+). In these quantifications, WT/GFP mice showed higher proportions of label-retaining cells than AD/GFP (mean ± SD: 0.13 ± 0.07% vs. 0.04 ± 0.04%; *n* = 11 and 10, respectively; *p* < 0.01), suggesting a reduction of the quiescent NSC pool over the course of AD. Remarkably, this deficit was reversed in AD/4D mice, which showed increased Sox2 + BrdU+/GFP + cells compared to AD/GFP mice (0.23 ± 0.16% vs. 0.04 ± 0.04%; *n* = 10 and 10, respectively; *p* < 0.01; Figure 1D) and reaching levels even higher, although not statistically different (*p* = 0.07), than WT/GFP mice (Figure 1D).

Finally, neurogenic output and/or neuronal survival was quantified as the proportion of BrdU+ cells expressing the neuronal marker NeuN among infected GFP + cells (i.e.: BrdU+NeuN+/GFP+) 8 weeks after BrdU labeling (i.e.: 16 weeks after 4D injection). Consistent with previous reports showing impaired adult neurogenesis in AD (Mu and Gage, 2011; Salta et al., 2023), WT/GFP mice showed higher levels of BrdU+NeuN+/GFP + adult-born neurons than AD/GFP mice (mean ± SD: 0.38 ± 0.16% vs. 0.16 ± 0.04%; n = 11 and 10, respectively; *p* < 0.001). Notably, AD/4D mice showed a significant increase in BrdU+NeuN+/GFP + cells compared with AD/GFP mice (0.51 ± 0.17% vs. 0.16 ± 0.04%; n = 10 and 10, respectively; *p* < 0.001), again reaching levels higher, although not statistically different (*p* = 0.19), than WT/GFP mice (Figure 1E).

Together, these data show that 4D retains its potential to promote NSC expansion even in the AD-compromised neurogenic niche, hence, restoring neurogenesis to levels comparable to WT healthy mice. In turn, this provides a basis to test whether enhancement of endogenous neurogenesis is accompanied by improvements in hippocampal-related behavior.

### 3.2 Enhanced neurogenesis attenuates hippocampal-related behaviors of 3xTg-AD mice

**Figure 2:**
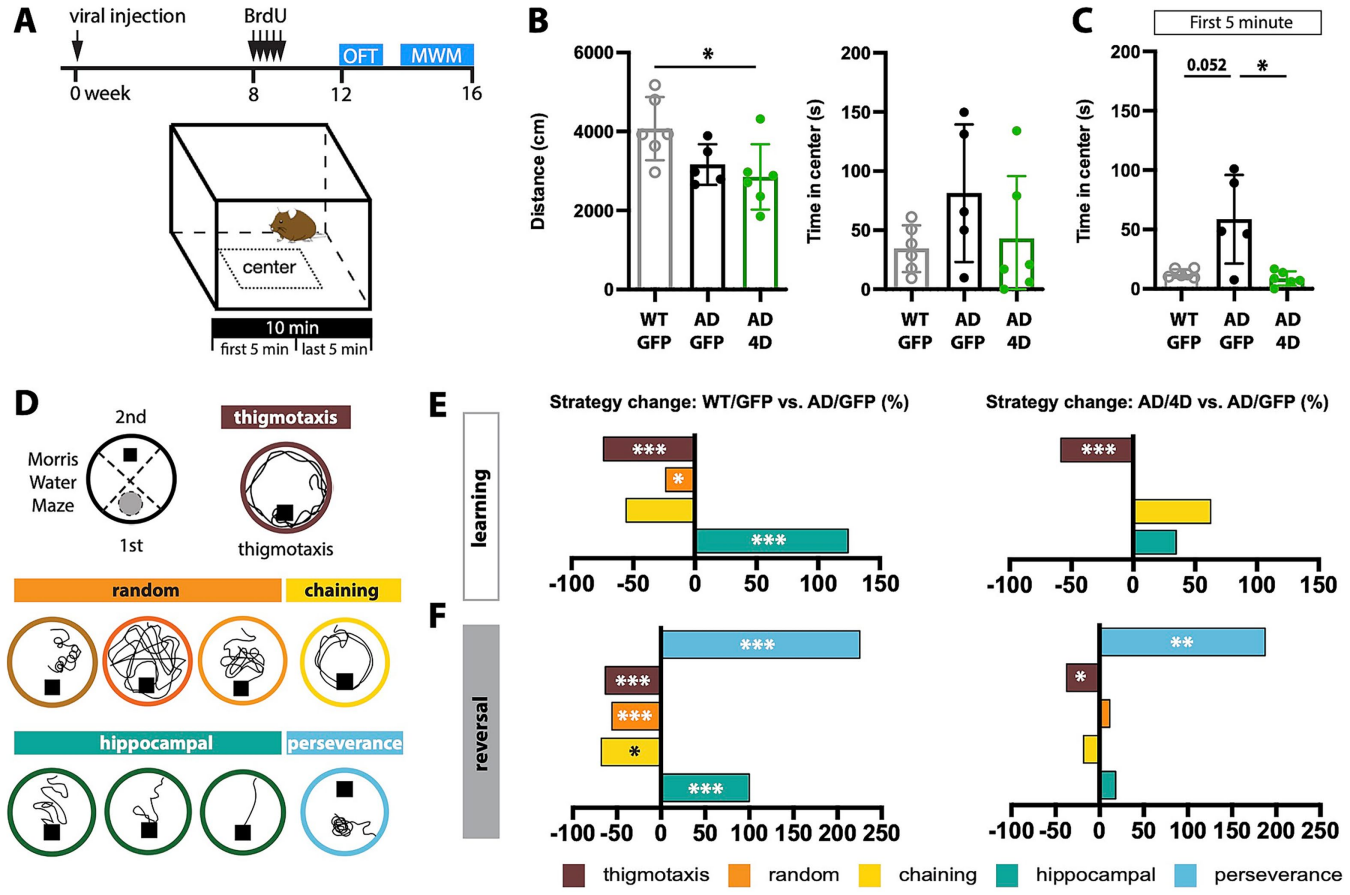
Enhancing neurogenesis attenuates selected hippocampal-related behavioral deficits of 3xTg-AD mice. **(A)** Schemes of the behavioral testing timeline and the open field test (OFT) setting. **(B)** Total distance travelled (cm) and time spent in the center zone (s). **(C)** Time spent in the center zone during the first 5-min interval of the OFT. Values are shown as bar graph with individual data points and mean ± SD (*n* = 6, 5, and 6 for WT/GFP, AD/GFP, and AD/4D, respectively). Pairwise comparisons in (B and C were performed using Student’s unpaired t-test, Welch’s unpaired t-test, or Mann–Whitney U test. **(D)** Representative MWM swim paths showing the navigational strategy categories and corresponding color codes: thigmotaxis, random strategies, egocentric chaining, hippocampal-dependent allocentric strategies, and perseverance upon reversal. **(E,F)** Percentage change in navigational strategy use during learning **(E)** and reversal **(F)**, comparing WT/GFP vs. AD/GFP (left) and AD/4D vs. AD/GFP (right). Values represent percentage change in strategy use relative to AD/GFP, calculated as [(strategy proportion in comparison group − strategy proportion in AD/GFP reference group)/strategy proportion in AD/GFP reference group] × 100. Strategy analyses were performed using logistic regression followed by Wald tests of odds ratios (*n* = 11, 13, and 13 for WT/GFP, AD/GFP, and AD/4D, respectively) * *p* < 0.05; ** *p* < 0.01; *** *p* < 0.001.

While improvements in NSC expansion and neurogenic output were encouraging, the critical question remains whether enhancing AHN translates into cognitive benefits in AD mice. To address this, we induced 4D overexpression as previously described and 12 weeks later subjected 3xTg-AD mice to the OFT and MWM (Figure 2A). These tests were chosen to assess open-field behavior in the absence of an explicit cognitive challenge and spatial navigation in a hippocampal-dependent task, respectively (Figures 2A,D).

Overall locomotor activity in the OFT showed limited group differences. Although AD/4D mice travelled less than WT/GFP mice (2,850 ± 828 cm vs. 4,074 ± 800 cm, *p* = 0.026; Figure 2B), we primarily focused on differences between WT/GFP vs. AD/GFP and AD/GFP vs. AD/4D cohorts, corresponding to AD-associated deficits and 4D-mediated, potential improvements in AD phenotypes, respectively. WT/GFP and AD/GFP mice did not differ significantly in total distance travelled (mean ± SD: 4,074 ± 800 cm vs. 3,162 ± 516 cm, *p* = 0.057; Figure 2B) with AD/GFP and AD/4D mice being also comparable (3,162 ± 516 cm vs. 2,850 ± 828 cm, *p* = 0.48; Figure 2B). Center duration over the full 10-min test did not differ significantly among groups (WT/GFP vs. AD/GFP: 34.3 ± 19.7 s vs. 81.3 ± 58.2 s, *p* = 0.15; AD/GFP vs. AD/4D: 81.3 ± 58.2 s vs. 42.9 ± 52.8 s, *p* = 0.28; WT/GFP vs. AD/4D: 34.3 ± 19.7 s vs. 42.9 ± 52.8 s, *p* = 0.72; Figure 2B).

To better dissect the nature of these non-significant trends in the center occupancy behavior, we increased the temporal resolution of our analysis by splitting the total duration of the test of 10 min into two intervals of 5 min each. During the first 5 min, WT/GFP mice showed a trend toward reduced center duration compared with AD/GFP mice (13.0 ± 3.5 s vs. 58.6 ± 37.4 s, *p* = 0.052; Figure 2C), whereas AD/4D mice spent significantly less time in the center than AD/GFP mice (8.8 ± 5.9 s vs. 58.6 ± 37.4 s, *p* = 0.040; Figure 2C) and were comparable to WT/GFP mice (8.8 ± 5.9 s vs. 13.0 ± 3.5 s, *p* = 0.16; Figure 2C). In addition, WT/GFP mice moved faster in the center than AD/GFP mice during the first 5 min (12.8 ± 2.6 cm/s vs. 6.9 ± 1.1 cm/s, *p* = 0.001; Supplementary Figure 2A), while AD/4D mice showed center speeds comparable to WT/GFP mice (13.0 ± 5.8 cm/s vs. 12.8 ± 2.6 cm/s, *p* = 0.95; Supplementary Figure 2A) and a trend toward increased center speed relative to AD/GFP mice (13.0 ± 5.8 cm/s vs. 6.9 ± 1.1 cm/s, *p* = 0.076; Supplementary Figure 2A). These differences were not detected during the last 5 min of the OFT (center duration, all comparisons *p* ≥ 0.81; center speed, all comparisons *p* ≥ 0.45; Supplementary Figure 2B). Together, these data suggest that 4D-mediated enhancement of neurogenesis is associated with partial normalization of early open-field center occupancy in AD mice, although these parameters may reflect a combination of exploration, anxiety-like behavior, locomotor activity, and risk assessment.

Next, we investigated whether 4D-enhanced neurogenesis was accompanied by changes in MWM performance, a hippocampal-dependent navigational task. MWM analyses were performed using mice from the OFT cohort (n = 6, 5, 6, respectively for WT/GFP, AD/GFP, and AD/4D) and an additional independent cohort (n = 5, 8, 7, respectively for WT/GFP, AD/GFP, and AD/4D). Analysis of standard MWM parameters, including pathlength, swimming speed, and escape latency, showed that WT/GFP mice travelled shorter distance, swam more slowly but faster to reach the platform than both AD/GFP and AD/4D mice (pathlength mean = 888, 1,554, and 1,506 cm; swimming speed mean = 17.1, 20.4, and 20.6 cm/s; escape latency mean = 50.5, 79.3, and 73.2 s for WT/GFP, AD/GFP, and AD/4D, respectively; Supplementary Figures 2C–E). In contrast, AD/GFP and AD/4D mice did not differ significantly in pathlength, swimming speed, or escape latency (Supplementary Figures 2C–E). Although AD/4D mice showed a non-significant reduction in escape latency compared with AD/GFP mice at selected time points, particularly during late learning and late reversal, this trend was not statistically significant (overall two-way ANOVA analysis, group effect, *p* = 0.53, Supplementary Figure 2E).

Despite the lack of differences in pathlength, speed and latency between AD/GFP and AD/4D mice, assessment of navigational strategies can help distinguish egocentric search patterns, hippocampal-dependent allocentric navigation and perseverance after reversal (Garthe and Kempermann, 2013; Grech et al., 2018), which may not be captured by standard MWM parameters alone. To perform these analyses, each trial was assigned one predominant strategy based on the swim trajectory and classified based on a previously adopted algorithm used in several previous reports (Berdugo-Vega et al., 2020; Overall et al., 2020; Schwab et al., 2022; Lehr et al., 2023): (i) thigmotaxis, (ii) random strategies, (iii) egocentric chaining, (iv) hippocampal-dependent allocentric strategies, and (v) perseverance upon reversal (see Material and Methods and Figure 2D).

When comparing the navigational strategies during 4 days of learning, WT/GFP mice progressed from thigmotaxis to random strategies and chaining to finally utilize mainly allocentric strategies more efficiently than AD/GFP mice (change in probability of WT/GFP vs. AD/GFP: hippocampal/allocentric = 124%, thigmotaxis = −74%; OR WT/GFP vs. AD/GFP: hippocampal/allocentric = 3.64, thigmotaxis = 0.21, Wald-test, *p* < 0.0001; Figure 2E, left and Supplementary Figure 2F). This was expected and corroborates the known impairment in learning characterizing the AD phenotype (Chen et al., 2000; Reiserer et al., 2007; Baeta-Corral and Giménez-Llort, 2015; Berkowitz et al., 2018). Notably, however, AD/4D mice showed an attenuation of the AD phenotypes with a reduced thigmotaxis (change in probability of AD/4D vs. AD/GFP: thigmotaxis = −58%; OR AD/4D vs. AD/GFP: thigmotaxis = 0.36, Wald-test, *p* < 0.0001) while displaying a trend, although not significant, toward the use of hippocampal-dependent allocentric strategies (change in probability of AD/4D vs. AD/GFP: hippocampal/allocentric = 34%; OR AD/4D vs. AD/GFP: hippocampal/allocentric = 1.51, Wald-test, *p* = 0.06; Figure 2E, right). After learning, the position of the platform was reversed and re-learning evaluated showing a reduced thigmotaxis and increased perseverance of AD/4D mice (change in probability of AD/4D vs. AD/GFP: thigmotaxis = −38%, perseverance = 187%; OR AD/4D vs. AD/GFP: thigmotaxis = 0.55, Wald-test, *p* < 0.05; perseverance = 4.36, Wald-test, *p* < 0.01; Figure 2F, right, and Supplementary Figure 2F).

Together, these results indicate that expansion of NSC by 4D overexpression attenuates some, but not other, behavioral deficits of 3xTg-AD mice supporting a partial, rather than complete, attenuation of hippocampal-related behavioral deficits upon enhancement of AHN.

## 4 Discussion

Our study shows that the AD-compromised neurogenic niche is still a susceptible target for genetic expansion of NSC and enhanced AHN despite ongoing neurodegeneration and amyloid plaque load. In past studies (Artegiani et al., 2011; Alonso et al., 2019; Berdugo-Vega et al., 2020), 4D overexpression was found to selectively increase NSC expansion and adult neurogenesis without any observable effect on mature neurons and glial cells. Although unknown additional effects of 4D cannot be formally excluded, our study supports the notion that enhanced AHN is accompanied by attenuation of certain cognitive deficits associated with AD, namely open-field center behavior and navigational strategy use.

Specifically, enhancing AHN attenuated selected AD-associated behaviors without fully restoring behavioral performance in standard MWM parameters, including pathlength, swimming speed, and escape latency. These behavioral findings should be interpreted cautiously and in the context of previously reported behavioral phenotypes in 3xTg-AD mice. For example, increased center occupancy in the OFT does not necessarily reflect increased exploration alone, but may also indicate altered anxiety-related behavior, disinhibition, habituation, locomotor activation, or risk assessment. Consistent with this interpretation, 6-month-old 3xTg-AD mice have been reported to show mild disinhibition in the OFT, reflected by increased time spent in the center early during testing (Hebda-Bauer et al., 2012). Similarly, the higher swimming speed observed in AD/GFP and AD/4D mice compared with WT/GFP mice may reflect altered behavioral activation, stress responsivity, motivation, or other non-cognitive components of MWM performance, consistent with reports of persistent hyperactivity and increased swimming speed in 3xTg-AD mice (Baeta-Corral and Giménez-Llort, 2015).

Therefore, we interpret the behavioral changes induced by 4D as a modulation of selected hippocampal-related measures rather than generalized rescue of exploration, anxiety, or spatial memory. Other hippocampal-specific cognitive functions, including novel object recognition, fear conditioning, context discrimination, etc., were not addressed in the current study. Whether findings using 6-month-old female 3xTg-AD mice could be generalized at a different age, sex, or other AD animal models remains to be assessed. Despite these limitations, our study provides additional evidence for AHN to be a valuable target for attenuation of deficits arising during AD progression. In this context, it is worth noting that the observed behavioral changes occurred despite unchanged amyloid plaque burden, suggesting that the effects of 4D were not driven by changes in plaque load. This dissociation has therapeutic implications, as AHN-targeted interventions may complement amyloid- and/or tau-directed therapies through distinct mechanisms.

Adult-born neurons are thought to contribute to hippocampal function by enhancing synaptic plasticity and refining memory precision (Anacker and Hen, 2017; Toda et al., 2019; Kempermann, 2022). The selected behavioral changes observed in AD/4D mice are consistent with these roles. Importantly, 4D not only drives proliferation of active NSC but also maintains quiescent NSC that are essential for the long-term regenerative potential of the neurogenic niche (Berdugo-Vega et al., 2020). This is an important consideration, as sustained neurogenesis is required to support cognitive function throughout life as well as over the course of long-lasting neurodegenerative diseases.

In summary, expansion of endogenous NSC could restore AHN during AD progression to levels comparable to the healthy brain and despite ongoing neurodegeneration. Increased AHN was accompanied by improvements in selected hippocampal-related behaviors highlighting a potential new avenue toward multi-modal therapeutic intervention in AD.

## Data Availability

The original contributions presented in the study are included in the article/Supplementary material, further inquiries can be directed to the corresponding author.
