# Supplementary material for: Enhancing adult neurogenesis attenuates hippocampal-related behavioral deficits in an Alzheimer’s mouse model
Source: Front Neurosci. 2026 Jun 22;20:1833016. doi: 10.3389/fnins.2026.1833016 (PMC13333594; doi:10.3389/fnins.2026.1833016)
Supplement: Supplementary file 1 [file Data_sheet_1.docx]

Supplementary Material

# Supplementary Figures and Tables


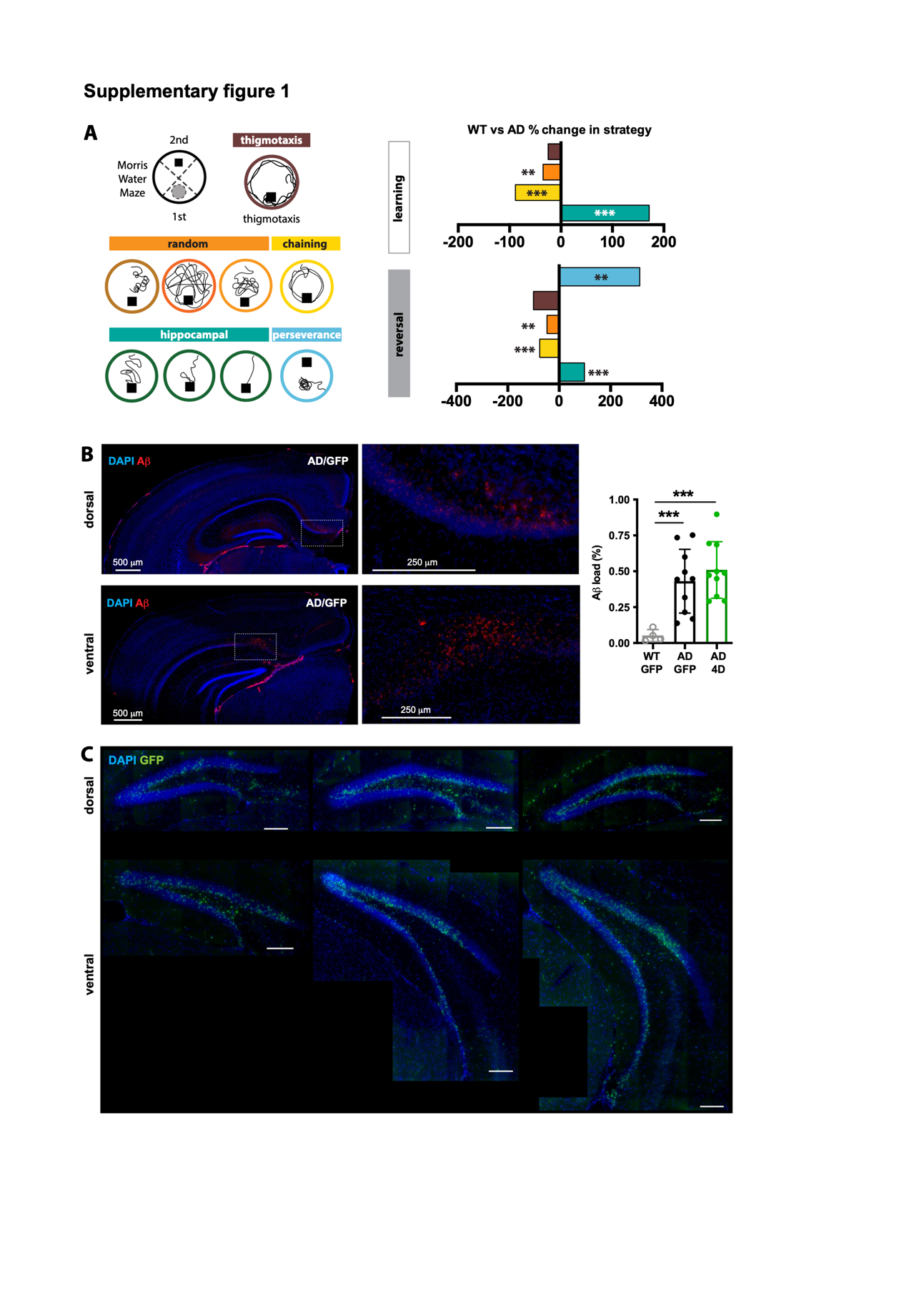


**Supplementary Figure 1. Analysis of AD phenotypes and pathologies in 3xTg AD mice.** **A**) Searching strategies assigned to each trial by the algorithm in MWM. Comparison of the percentage change in searching strategies relative to 3xTg-AD across trials during the learning and reversal phase between WT and 3xTg-AD at 6-month-old. (n = 8 and 6 for WT and 3xTg-AD groups respectively), Wald-test, ** *p* < 0.01, *** *p* < 0.001. **B**) Representative Aβ staining image and quantification of Aβ levels in the hippocampus. Values are shown as bar graph with individual data points and mean ± SD (n = 4, 10, and 10 for WT/GFP, AD/GFP, and AD/4D groups respectively), Pairwise comparisons used Student’s unpaired t-test, Welch’s unpaired t-test, or Mann–Whitney U test as appropriate, *** *p* < 0.001. Values of mean ± SD are listed in Supplementary Table 2. **C**) Representative rostro-caudal hippocampal sections from an AD/4D mouse showing the distribution of GFP+ virally infected cells in the dentate gyrus. Scale bar = 100 μm.

**
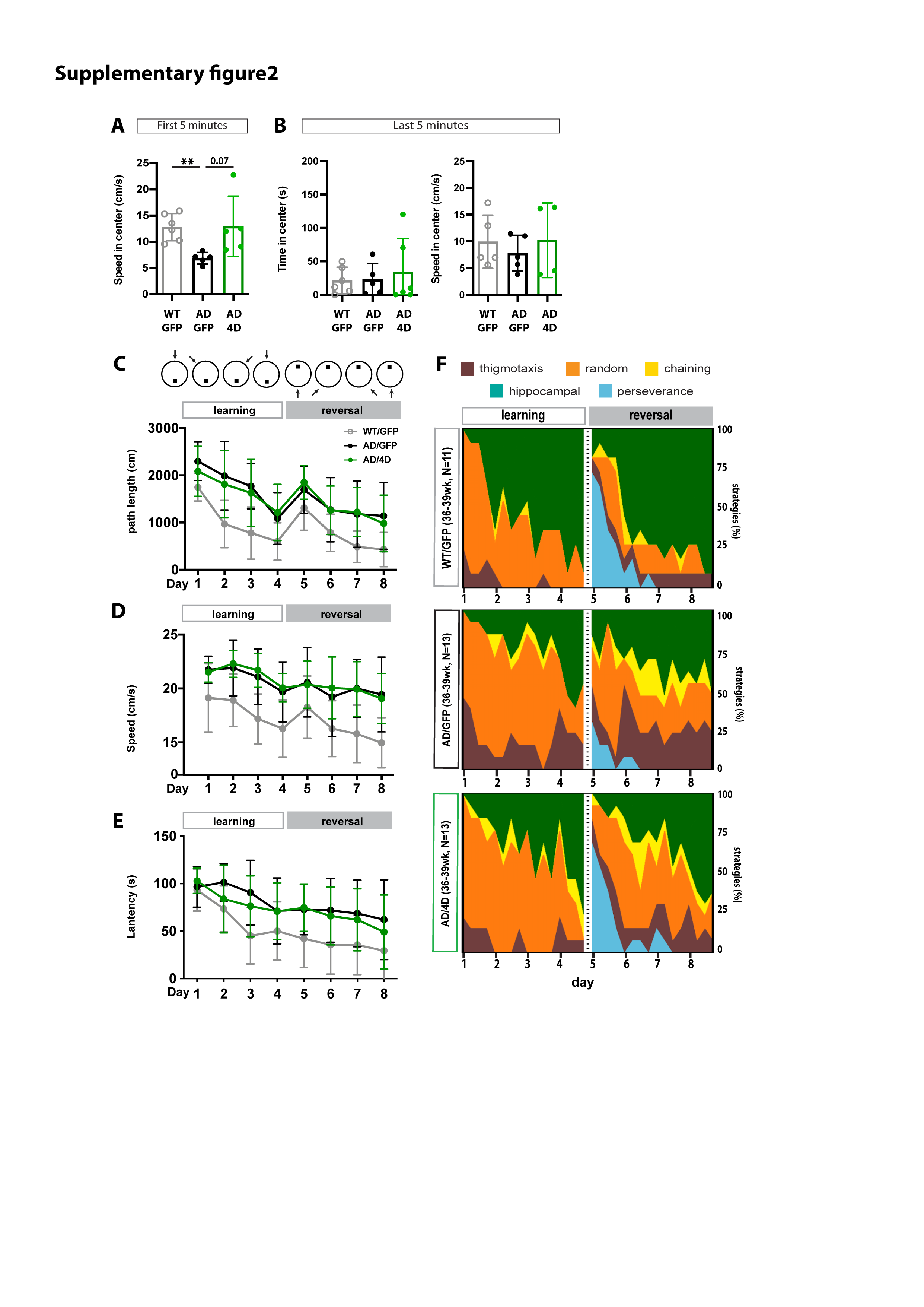
**

**Supplementary Figure 2. Additional analysis in navigational performance of WT, AD/GFP and AD/4D mice.** **A**) Speed in the center (cm/s) of the open field during the initial 5-minute exploration. **B**) Time (s) and speed (cm/s) in the center of the open field during the last 5-minute exploration. **A**) – **B**) Values are shown as bar graph with individual data points and mean ± SD (n = 6, 5, and 6 for WT/GFP, AD/GFP, and AD/4D groups respectively), Pairwise comparisons used Student’s unpaired t-test, Welch’s unpaired t-test, or Mann–Whitney U test as appropriate, * *p* < 0.05, ** *p* < 0.01. Values are listed in Supplementary Table 3. **C**) Depiction of starting points in MWM and swimming path length (cm) in MWM. A significant group difference was observed between WT/GFP and AD/GFP (two-way ANOVA, time F (7, 154) = 25.39, *p* < 0.0001, group F (1,22) = 20.74, *p* < 0.001, interaction F (7, 154) = 1.890, *p* = 0.07). No difference was observed between AD/4D and AD/GFP (two-way ANOVA, time F (7, 168) = 22.09, *p* < 0.0001, group F (1, 24) = 0.08419, *p* = 0.77, interaction F (7, 168) = 0.6700, *p* = 0.69). **D**) Swimming speed (cm/s) in MWM. A significant group difference was observed between WT/GFP and AD/GFP (two-way ANOVA, time F (7, 154) = 11.31, *p* < 0.0001, group F (1, 22) = 13.91, *p* < 0.001, interaction F (7, 154) = 1.109, *p* = 0.36). No difference was observed between AD/4D and AD/GFP (two-way ANOVA, time F (7, 168) = 8.053, *p* < 0.0001, group F (1, 24) = 0.05675, *p* = 0.081, interaction F (7, 168) = 0.3622, *p* = 0.92). **E**) Latency to platform (s) in MWM. A significant group difference was observed between WT/GFP and AD/GFP (two-way ANOVA, time F (7, 154) = 19.67, *p* < 0.001, group F (1, 22) = 8.21, *p* < 0.01, interaction F (7, 154) = 2.45, *p* < 0.05). No difference was observed between AD/4D and AD/GFP (two-way ANOVA, time F (7, 168) = 13.49, *p* < 0.001, group F (1, 24) = 0.4, *p* = 0.53, interaction F (7, 168) = 1.1, *p* = 0.36). **F**) Schematics, color code, and the contribution of swimming strategies as percentages. **C**) – **F**) For WT/GFP, AD/GFP, and AD/4D groups, the numbers of mice were indicated (n=11, 13, and 13 respectively).

**Supplementary Table 1. Primary and secondary antibodies for immunohistochemistry.**

| **Primary antibodies** | | | |
| --- | --- | --- | --- |
| **Antigen** | **Dilution** | **Supplier** | **Catalog number** |
| BrdU | 1:250 | Abcam | ab6326 |
| Sox2 | 1:500 | Santa Cruz Biotechnology | Sc-17320 |
| S100β | 1:1000 | Abcam | ab14688 |
| DCX | 1:100 | Abcam | ab18723 |
| NeuN (Fox3) | 1:1000 | Abcam | ab104225 |
| GFP | 1:1000 | Abcam | Ab13970 |
| Aβ | 1:1500 | Novus Biologicals | NBP2-13075 |
| **Secondary antibodies:** IgG raised in donkey (against goat, mouse, rabbit, rat and guinea pig), conjugated to different fluorophores (DyLight, Alexa or Cyanines), all purchased from Jackson Immunoresearch. | | | |

**Supplementary Table 2. Comparison of navigational performance, amyloid plaque load in the hippocampus and cell quantifications.**

| **Odds ratio of navigational strategy in the learning phase MWM (WT vs. AD)** | | | | | | | | | | | | | | | | | | | | | | | | |
| --- | --- | --- | --- | --- | --- | --- | --- | --- | --- | --- | --- | --- | --- | --- | --- | --- | --- | --- | --- | --- | --- | --- | --- | --- |
| **Thigmotaxis** | | | **Random** | | | | | | | **Chaining** | | | | | | | | | **Hippocampal** | | | | | |
| 0.75 | | | 0.46 | | | | | | | 0.09 | | | | | | | | | 5.22 | | | | | |
| **Odds ratio of navigational strategy in the reversal phase MWM (WT vs. AD)** | | | | | | | | | | | | | | | | | | | | | | | | |
| **Thigmotaxis** | | **Random** | | | **Chaining** | | | | | | | **Hippocampal** | | | | | | **Perseverance in reversal phase 1^st^ day** | | | | | | |
| 0 | | 0.43 | | | 0.18 | | | | | | | 5.21 | | | | | | 5.6 | | | | | | |
| **Hippocampal Aβ load (%)** | | | | | | | | | | | | | | | | | | | | | | | | |
| WT/GFP (n = 4) | | 0.0519 ± 0.0421 | | | | | | | | | | | | | | | | | | | | | | |
| AD/GFP (n = 10) | | 0.4308 ± 0.2218 | | | | | | | | | | | | | | | | | | | | | | |
| AD/4D (n =10) | | 0.5094 ± 0.1970 | | | | | | | | | | | | | | | | | | | | | | |
| **Cell quantifications of individual mouse** | | | | | | | | | | | | | | | | | | | | | | | | |
| **BrdU+/Sox2+GFP+ (%)** | | | | | | | | | | | | | | | | | | | | | | | | |
| AD/GFP (n = 4) | | 1.75 | | | | 2.53 | | | | | 2.87 | | | | | 2.00 | | | | | |  | | |
| AD/4D (n = 5) | | 2.67 | | | | 3.47 | | | | | 5.33 | | | | | 4.17 | | | | | | 3.03 | | |
| **Sox2+S100β–/GFP+ (%)** | | | | | | | | | | | | | | | | | | | | | | | | |
| WT/GFP (n = 11) | | 13.12 | | 11.55 | 8.35 | | | 8.25 | 8.92 | | | | 9.04 | | 5.97 | | 10.17 | | | 6.42 | | | 5.62 | 9.02 |
| AD/GFP (n = 10) | | 13.77 | | 11.18 | 14.79 | | | 10.50 | 7.50 | | | | 7.64 | | 3.57 | | 6.35 | | | 4.32 | | | 6.00 |  |
| AD/4D (n = 10) | | 18.59 | | 17.88 | 14.20 | | | 17.36 | 18.67 | | | | 12.34 | | 7.54 | | 10.83 | | | 16.36 | | | 9.67 |  |
| BrdU+Sox2+/GFP+ (%) | | | | | | | | | | | | | | | | | | | | | | | | |
| WT/GFP (n = 11) | | 0.17 | | 0.11 | 0.2 | | | 0.00 | 0.19 | | | | 0.06 | | 0.18 | | 0.16 | | | 0.09 | | | 0.08 | 0.18 |
| AD/GFP (n = 10) | | 0.09 | | 0.06 | 0.00 | | | 0.05 | 0.00 | | | | 0.06 | | 0.00 | | 0.06 | | | 0.12 | | | 0.00 |  |
| AD/4D (n = 10) | | 0.58 | | 0.17 | 0.34 | | | 0.29 | 0.2 | | | | 0.06 | | 0.04 | | 0.31 | | | 0.21 | | | 0.14 |  |
| **BrdU+NeuN+/GFP+ (%)** | | | | | | | | | | | | | | | | | | | | | | | | |
| WT/GFP (n = 11) | | 0.23 | | 0.21 | 0.29 | | | 0.47 | 0.60 | | | | 0.32 | | 0.24 | | 0.62 | | | 0.59 | | | 0.23 | 0.44 |
| AD/GFP (n = 10) | | 0.14 | | 0.14 | 0.14 | | | 0.14 | 0.17 | | | | 0.18 | | 0.21 | | 0.22 | | | 0.13 | | | 0.10 |  |
| AD/4D (n = 10) | | 0.52 | | 0.86 | 0.23 | | | 0.43 | 0.45 | | | | 0.67 | | 0.39 | | 0.54 | | | 0.52 | | | 0.47 |  |
| **Neurogenesis level of groups** | | | | | | | | | | | | | | | | | | | | | | | | |
| **Group** | **BrdU+ / Sox2+ GFP+ (%)** | | | | | | **Sox2+S100β– / GFP+ (%)** | | | | | | | **BrdU+Sox2+ / GFP+ (%)** | | | | | | | **BrdU+NeuN+ / GFP+ (%)** | | | |
| WT/GFP | N/A | | | | | | 8.766 ± 2.291 | | | | | | | 0.1291 ± 0.0650 | | | | | | | 0.3840 ± 0.1619 | | | |
| AD/GFP | 2.287 ± 0.503 | | | | | | 8.562 ± 3.842 | | | | | | | 0.0440 ± 0.0427 | | | | | | | 0.1582 ± 0.0369 | | | |
| AD/4D | 3.733 ± 1.052 | | | | | | 14.34 ± 4.041 | | | | | | | 0.2340 ± 0.1572 | | | | | | | 0.5071 ± 0.1698 | | | |

Odds ratios (OR) of MWM strategy comparisons between WT and 3xTg-AD mice, hippocampal Aβ load, and individual mouse-level cell and group quantification values, corresponding to Fig. 1 and Fig. S1. N/A, not applicable.

**Supplementary Table 3. Behavioral analysis of the OFT and MWM.**

| **Open field test** | | | | | | | | | | | | |
| --- | --- | --- | --- | --- | --- | --- | --- | --- | --- | --- | --- | --- |
| **Group** | **Travel distance (cm)** | | | **Speed (cm/s)** | | | | **Center duration in 10 min (s)** | | **Center duration in the first 5 min (s)** | | |
| WT/GFP | 4074 ± 800 | | | 6.79 ± 1.33 | | | | 34.34 ± 19.70 | | 12.98± 3.51 | | |
| AD/GFP | 3162 ± 516 | | | 5.27 ± 0.86 | | | | 81.31 ± 58.17 | | 58.55 ± 37.36 | | |
| AD/4D | 2850 ± 828 | | | 4.75 ± 1.38 | | | | 42.87 ± 52.83 | | 8.76 ± 5.93 | | |
| **Group** | **Center speed in the first 5 min (cm/s)** | | | | | **Center duration in the last 5 min (s)** | | | | **Center speed in the last 5 min (cm/s)** | | |
| WT/GFP | 12.81 ± 2.60 | | | | | 21.36 ± 19.90 | | | | 9.94 ± 4.96 | | |
| AD/GFP | 6.86 ± 1.09 | | | | | 22.73 ± 23.99 | | | | 7.80 ± 3.32 | | |
| AD/4D | 12.96 ± 5.75 | | | | | 34.09 ± 50.09 | | | | 10.22 ± 6.97 | | |
| **Odds ratio of navigational strategy in the learning phase MWM (WT/GFP vs. AD/GFP)** | | | | | | | | | | | | |
| **thigmotaxis** | | | **random** | | | | **chaining** | | | | **hippocampal** | |
| 0.22 | | | 0.6 | | | | 0.43 | | | | 3.64 | |
| **Odds ratio of navigational strategy in the reversal phase MWM (WT/GFP vs. AD/GFP)** | | | | | | | | | | | | |
| **thigmotaxis** | | **random** | | | **chaining** | | | | **hippocampal** | | | **perseverance in reversal phase 1^st^ day** |
| 0.3 | | 0.36 | | | 0.3 | | | | 5 | | | 5.5 |
| **Odds ratio of navigational strategy in the learning phase MWM (AD/4D vs. AD/GFP)** | | | | | | | | | | | | |
| **thigmotaxis** | | | **random** | | | | **chaining** | | | | **hippocampal** | |
| 0.36 | | | 0.98 | | | | 1.67 | | | | 1.51 | |
| **Odds ratio of navigational strategy in the reversal phase MWM (AD/4D vs. AD/GFP)** | | | | | | | | | | | | |
| **thigmotaxis** | | **random** | | | **chaining** | | | | **hippocampal** | | | **perseverance in reversal phase 1^st^ day** |
| 0.55 | | 1.17 | | | 0.8 | | | | 1.32 | | | 4.36 |

Group and mean ± SD of OFT travel distance, speed and time spent in the center, corresponding to Fig. 2B, 2C, S2A, and S2B. Odds ratio (OR) of swimming strategy in learning and reversal phase comparing between groups, corresponding to Fig. 2E, F.
